# Association of low plasma antioxidant levels with all-cause mortality and coronary events in healthy middle-aged men from France and Northern Ireland in the PRIME study

**DOI:** 10.1007/s00394-020-02455-2

**Published:** 2020-12-23

**Authors:** Gareth J. McKay, Natalie Lyner, Gerry J. Linden, Frank Kee, Marie Moitry, Katia Biasch, Philippe Amouyel, Jean Dallongeville, Vanina Bongard, Jean Ferrières, K. Fred Gey, Chris C. Patterson, Jayne V. Woodside

**Affiliations:** 1 Centre for Public Health, Royal Victoria Hospital, Queen’s University Belfast, Belfast, BT12 6BA UK; 2 MONICA-Strasbourg, Strasbourg, France; 3 MONICA-Lille, Lille, France; 4 MONICA-Toulouse, Toulouse, France; 5 The MONICA Reference Centre for Vitamins, Department of Biochemistry and Molecular Biology, University of Bern, Bern, Switzerland

**Keywords:** Cardiovascular disease, Premature mortality, Antioxidant, Vitamin, Carotenoid, Retinol

## Abstract

**Background:**

The main underlying risk factors associated with coronary heart disease (CHD) are modifiable and oxidative injury and systemic inflammatory damage represent key aetiological factors associated with the development and progression of CHD and premature mortality.

**Objective:**

To examine associations of plasma antioxidant status with all-cause mortality and fatal or non-fatal cardiovascular events.

**Design:**

The PRIME study prospectively evaluated 9709 men aged 50–59 years between 1991 and 1993 in Northern Ireland and France who were free of CHD at recruitment and followed annually for deaths and cardiovascular events for 10 years. Serum concentrations of vitamin C, retinol, two forms of vitamin E (α- and γ-tocopherol) and six carotenoids were quantified by high-performance liquid chromatography. Baseline conventional risk factors were considered, as well as socioeconomic differences and lifestyle behaviours including diet, smoking habit, physical activity, and alcohol consumption through Cox regression analyses.

**Results:**

At 10 years, there were 538 deaths from any cause and 440 fatal or non-fatal cardiovascular events. After adjustment for country, age, systolic blood pressure, diabetes, body mass index, cholesterol, high density lipoprotein cholesterol, triglycerides, height, total physical activity, alcohol consumption and smoking habit, higher levels of all antioxidants were associated with significantly lower risk of all-cause mortality, with the exception of γ-tocopherol. Only retinol was significantly associated with decreased risk of cardiovascular events in a fully adjusted model.

**Conclusions:**

Low antioxidant levels contribute to the gradient of all-cause mortality and cardiovascular incidence independent of lifestyle behaviours and traditional cardiovascular and socioeconomic risk factors.

**Supplementary Information:**

The online version contains supplementary material available at 10.1007/s00394-020-02455-2.

## Introduction

Cardiovascular disease (CVD) is the leading global cause of death accounting for an estimated 17.9 million deaths in 2016 according to the World Health Organisation. This represents 31% of all deaths worldwide, a figure expected to increase with rising obesity and diabetes prevalence, leading to a greater burden on healthcare provision [1]. The main underlying risk factors associated with CVD are modifiable and evidence has implicated oxidative injury and systemic inflammatory damage as key aetiological factors in both the development and progression of CVD pathogenesis [2–4]. A high intake of fruit, vegetables and nuts has been associated with a reduction in CVD risk and premature mortality [5–7], with nutrition and diet estimated to account for approximately 45% of all adult cardiometabolic deaths in the United States in 2012 [8]. Biomarker measurement provides improvements over estimates of nutritional intake, strengthening the reliability of association studies in nutritional epidemiology [9]. Nevertheless, the influence of dietary antioxidants on CVD risk and all-cause mortality remains unclear [10]. LDL-cholesterol becomes more atherogenic following oxidative modification, leading to its accumulation on arterial walls, while antioxidants have been shown to reduce the rate of progression of atherosclerosis [11, 12]. Fat-soluble antioxidants such as vitamins A and E, as well as the serum carotenoids, are carried in the fatty core of LDL particles, prolonging LDL resistance to oxidative damage [12]. Plausible mechanisms by which nutritional factors, such as serum carotenoids and vitamins, may reduce CVD risk and premature mortality, have been proposed, comprising anti-oxidative and/or anti-inflammatory processes that counteract the effects of free radicals, including those common to vascular and non-vascular diseases [13]. Nevertheless, randomised supplementation trials that are largely limited to single antioxidant evaluation, have failed to consistently report reduced risk associated with CVD events and all-cause mortality identified through observational epidemiological studies, with some reporting unanticipated harmful effects [14]. While reduced all-cause mortality has been reported in nutritionally deficient individuals with low plasma antioxidant levels following supplementation, excess levels in sufficient individuals may not offer additional benefit and possibly result in potentially toxic effects [15–17].

Contrasting lifestyle and dietary behaviours, including antioxidant bioavailability, may contribute to variation in CVD risk and all-cause mortality as previously reported for the higher incidence of ischaemic heart disease in Northern Ireland (NI) compared to France [18, 19]. We hypothesised that variation in plasma retinol, alpha- and gamma-tocopherol (referred to as antioxidant vitamins), vitamin C, as well as the six major serum-based carotenoids: lutein, zeaxanthin, beta-cryptoxanthin, lycopene, alpha- and beta-carotene may be associated with cardiovascular and mortality outcomes in participants from the Prospective Epidemiological Study of Myocardial Infarction (PRIME). The PRIME study prospectively examined middle-aged men in France and NI, countries with contrasting culture and lifestyle. The aim of this study was to evaluate associations between levels of exogenous antioxidants in the plasma of study participants characterised as free from CVD at the point of recruitment with the incidence of coronary events and all-cause mortality over a 10-year follow-up period.

## Methods

### Participants

The PRIME Study is a multi-centre, prospective cohort composed of men aged 50–59 years when recruited from the general population of Belfast in NI and Lille, Strasbourg and Toulouse in France, between 1991 and 1993 [20, 21]. A detailed medical, lifestyle and food frequency questionnaire (FFQ) was completed at baseline examination which included information on lifetime smoking, physical activity [22], alcohol consumption [23] and diet [24]. A cardiovascular screening examination was also conducted at baseline, which included a detailed history of previous CVD. Follow-up included annual questionnaires for 10 years and possible cardiovascular events were confirmed by a medical committee [21, 25]. Of the 10,600 men recruited, 317 (3.0%) were lost to follow-up after 10 years, 215 (2%) withdrew their consent for participation within the study, and 653 (6.2%) died during this time. Cardiovascular events included validated myocardial infarction and/or stroke (fatal and non-fatal). Death certificates were obtained for all men who died and causes of death were categorised using the International Classification of Diseases (ICD) Ninth Revision.

### Serum antioxidant measurement

Fasting venous blood samples were drawn at baseline and centrifuged within 4 h of collection. Plasma samples were aliquoted for long-term storage at − 80 °C. Plasma concentrations of retinol, α-tocopherol, γ-tocopherol, and six carotenoids (α-carotene, β-carotene, β-cryptoxanthin, lutein, lycopene, zeaxanthin) were determined by high performance liquid chromatography (HPLC) with diode array detection [26]. Results were analysed using Chromquest 4.2 software (Thermo Fisher Scientific, Massachusetts, USA). Standards used in the assay were supplied by either Sigma (Sigma-Aldrich, Poole, Dorset, UK) or Chemos (Chemos GmbH, Regensburg, Germany). Vitamin C was measured in plasma which had been stabilised on collection on a BMG FLUOstar Optima plate reader adapted from the method by Vuilleumier and Keck [27]. These assays were validated against the National Institute of Standards and Technology (NIST) Standard Reference Material 968d for fat-soluble vitamins, carotenoids and cholesterol and NIST Standard Reference Material 970 for ascorbic acid in human serum, which were externally quality assured three times per year using samples supplied by the French Society for Vitamins and Biofactors. In-house quality control samples were also included in every run. The inter- and intra-assay coefficients of variation (CV) for vitamin A, E and carotenoid assays were < 15% and < 7.5% for vitamin C. A mean standardised antioxidant score (SAS) was estimated from the mean standardised *Z* scores for the ten logarithmically transformed antioxidants.

### Lifestyle variables

Body mass index (BMI) was calculated as the body weight/height^2^ (kg/m^2^). Lifetime smoking was expressed in five categories: never smoked, smoked other than cigarettes, smoked < 15 cigarette pack years, smoked ≥ 15 but < 30 cigarette pack years and smoked ≥ 30 cigarette pack years. Alcohol intake was also expressed as five categories of consumption; none, 1–128, 129–265, 266–461 and ≥ 462 mL/week. Physical activity (in metabolic equivalent of task (METs) scores/week) was positively skewed and was therefore summarised using median and interquartile range. Diabetes was defined by self-report. Socioeconomic status was categorised into high, middle and low using a composite measure of material conditions based on the type of living accommodation (rented or owned, mortgaged), number of cars/vans/motorcycles in the household and the number of baths and/or showers and toilets in the home [22].

The daily fruit, vegetable and juice (FVJ) score included all fruit and vegetable variables from the PRIME FFQ. This involved eight categories: ‘salad’, ‘carrot’, ‘tomato’, ‘natural’ and ‘baked vegetables’, ‘citrus fruits’, ‘other fruits’ and a ‘juice’ variable. Potatoes were not included as they were included under the ‘Bread, cereals and potatoes’ food group. Frequencies of each variable were transformed into daily consumption values (the ‘juice’ variable was limited to a maximum of one daily portion in-line with UK dietary guidelines) and these new variables were summed for each participant to give total daily FVJ consumption. This variable was positively skewed and was therefore summarised using median and interquartile range.

Ethical approval for baseline examination and follow-up was obtained at each study centre; all participants provided informed consent and the study was compliant with the Declaration of Helsinki guidelines.

### Statistical methods

Data for antioxidant levels were not normally distributed and were therefore logarithmically transformed before analysis. Results were summarised using the geometric mean and inter-quartile range. Correlation coefficients between fruit and vegetable consumption and antioxidant measures were assessed. Antioxidant levels were compared between men with and without a CVD outcome and all-cause mortality using independent samples t-tests. Lifestyle behaviours were compared across socioeconomic variables either by chi-square test for categorical variables or by Kruskal–Wallis one way ANOVA for continuous variables.

Cox proportional hazards models were used for analysis of antioxidant status for cardiovascular outcomes (first cardiovascular event) and separately for all-cause mortality, during a 10-year follow-up period. In the analysis of cardiovascular events, individuals were followed from baseline assessment to the first CVD event or death or end of follow-up. Each antioxidant was split into fourths using quartiles to categorise participants for analysis. The lowest quarter of the distribution of each antioxidant was used as the reference category for the calculation of hazard ratios. Firstly, models were run with minimal adjustment for potential confounders which included age and country, followed by a multivariate analysis that also included systolic blood pressure (SBP), diabetes, BMI, total cholesterol, high density lipoprotein (HDL) cholesterol, height, social class (low, medium and high), alcohol consumption (none, 1–128, 129–265, 266–461 and ≥ 462 mL/week), smoking status (never smoked, smoked other than cigarettes, smoked < 15 cigarette pack years, smoked ≥ 15 but < 30 cigarette pack years and smoked ≥ 30 cigarette pack years), and total physical activity (total physical activity was square root transformed, before inclusion in the model). Hazard ratios were compared across quarters of antioxidant distribution by a test for trend, and a likelihood ratio test was used to check for non-linearity by comparing the trend model with a model specifying a different hazard for each quarter. Additional sensitivity analyses considered the exclusion of variables that could potentially contribute to the causal pathway of CVD or all-cause mortality. Tests of the hazard proportionality assumption in the Cox model were performed using Schoenfeld residuals. All tests were conducted at the *p* < 0.05 significance level using SPSS version 25 (IBM Corp, Armonk, NY, USA) and Stata release 14 (StatCorp, College Station, TX, USA). To check for possible effect modification, interactions between country and antioxidants were added to the Cox proportional hazards models. A more stringent level of significance was set at *p* ≤ 0.005 to allow for multiple comparisons of potential interactions in the absence of any prior hypotheses.

## Results

### Cardiovascular disease events

Of the 10,600 PRIME study participants, 891 had a previous history of CVD at study entry (355 from NI and 536 from France) and were excluded from the analysis. The remaining 9709 men were followed up for 10 years (mean = 3506.81 days) which equated to 93,281 person years. Follow-up was incomplete for 470 men (the number who were not known to have died and were not followed for the full 10 years), so the lost to follow-up rate was 4.8%. Of 9709 men, there were 538 deaths from any cause and 440 fatal and non-fatal cardiovascular events after 10 years of follow-up. Population characteristics for those 9709 men are summarised according to CVD outcome and all-cause mortality (Table 1). Individuals who experienced a CVD outcome were on average older than men who had not (55.5 years versus 54.8 years), were more likely to be from Northern Ireland (38.4% versus 24.0%), to have diabetes (7.3% versus 3.1%) and higher mean SBP (141 mmHg versus 133 mmHg). In addition, those who experienced a CVD outcome had higher mean BMI (27.3 kg/m^2^ versus 26.5 kg/m^2^), higher mean total cholesterol (2.30 g/L versus 2.21 g/L) and median triglycerides (1.44 g/L versus 1.21 g/L) but lower mean HDL cholesterol (0.45 g/L versus 0.49 g/L), and height (172.1 cm versus 172.8 cm) and be less physically active (median METs 76.0 versus 83.2). Furthermore, they were more likely to be from the lowest social class (31.0% versus 23.8%), consume fewer daily FVJ portions (median 2.21 versus 2.43), be heavy smokers (31.3% versus 21.5%) and refrain from alcohol consumption (25.9% versus 16.3%).

**Table 1:** Summary characteristics for cardiovascular disease outcomes and all-cause mortality for 9709 men from Northern Ireland and France who were free of cardiovascular disease at entry

Parameter	CVD outcome			All-cause mortality		
	No	Yes	*p* value	No	Yes	*p* value
Age (years), mean (SD)	54.8 (2.9)	55.5 (3.0)	< 0.001	54.8 (2.9)	55.8 (2.8)	< 0.001
Country, *n* (%)						
Northern Ireland	2221 (24.0%)	169 (38.4%)		2219 (24.2%)	171 (31.8%)	
France	7048 (76.0%)	271 (61.6%)	< 0.001	6952 (75.8%)	367 (68.2%)	< 0.001
Diabetes, *n* (%)						
Yes	290 (3.1%)	32 (7.3%)		287 (3.1%)	35 (6.5%)	
No	8979 (96.9%)	408 (92.7%)	< 0.001	8884 (96.9%)	503 (93.5%)	< 0.001
Systolic blood pressure (mmHg), mean (SD)	133 (19)	141 (22)	< 0.001	133 (19)	139 (22)	< 0.001
Body mass index (kg/m^2^), mean (SD)	26.5 (3.4)	27.3 (3.8)	< 0.001	26.6 (3.38)	26.5 (4.23)	0.64
Total cholesterol (g/L), mean (SD)	2.21 (0.38)	2.30 (0.39)	< 0.001	2.22 (0.38)	2.19 (0.44)	0.21
HDL cholesterol (g/L), mean (SD)	0.49 (0.13)	0.45 (0.12)	< 0.001	0.49 (0.13)	0.49 (0.15)	0.72
Triglycerides (g/L), median (IQR)	1.21 (0.90–1.71)	1.44 (1.06–2.06)	< 0.001	1.22 (0.90–1.71)	1.31 (0.93–1.91)	0.008
Height (cm), mean (SD)	172.8 (6.6)	172.1 (6.4)	0.04	172.8 (6.5)	172.3 (6.7)	0.08
Total physical activity (MET),median (IQR)	83.2 (43–135)	76.0 (33.1–132)	0.03	83.3 (42.9–135)	73.6 (36.6–136)	0.04
Daily fruit, vegetable and juice portions, median (IQR)	2.43 (1.64–3.36)	2.21 (1.39–3.00)	< 0.001	2.43 (1.64–3.36)	2.14 (1.39–3.14)	< 0.001
Social class, *n* (%)						
Low	2201 (23.8%)	136 (31.0%)		2137 (23.4%)	200 (37.3%)	
Middle	1396 (15.1%)	76 (17.3%)		1403 (15.4%)	69 (12.9%)	
High	5638 (61.1%	227 (51.7%)	< 0.001	5598 (61.3%)	267 (49.8%)	< 0.001
Alcohol, *n* (%)						
None	1513 (16.3%)	114 (25.9%)		1527 (16.7%)	100 (18.6%)	
1–128 mL/week	1997 (21.5%)	78 (17.7%)		1983 (21.6%)	92 (17.1%)	
129–265 mL/week	1981 (21.4%)	92 (20.9%)		1974 (21.5%)	99 (18.4%)	
266–461 mL/week	1873 (20.2%)	71 (16.1%)		1845 (20.1%)	99 (18.4%)	
≥ 462 mL/week	1905 (20.6%)	85 (19.3%)	< 0.001	1842 (20.1%)	148 (27.5%)	< 0.001
Smoking, *n* (%)						
Never	2763 (30.0%)	111 (25.5%)		2765 (30.3%)	109 (20.3%)	
Smoked other than cigarettes	702 (7.6%)	27 (6.2%)		696 (7.6%)	33 (6.2%)	
Smoked < 15 pack years	2008 (21.8%)	66 (15.2%)		1999 (21.9%)	75 (14.0%)	
Smoked ≥ 15 but < 30 pack years	1755 (19.1%)	95 (21.8%)		1750 (19.2%)	100 (18.7%)	
Smoked ≥ 30 pack years	1984 (21.5%)	136 (31.3%)	< 0.001	1901 (20.9%)	219 (40.9%)	< 0.001

*MET* metabolic equivalent of task (per week), *SD* standard deviation, *IQR* interquartile range

### Antioxidant concentrations

Antioxidant status was available for 6961 participants and antioxidant quartile boundaries are shown in Supplementary Table 1. As expected, there was a high correlation between fruit and vegetable consumption and antioxidant measures (Supplementary Table 2). Over a third of participants (37.6%) had plasma vitamin C concentrations considered to be optimum for health (> 50 μmol/L), over three quarters (77.6%) had plasma vitamin C concentrations above the marginal deficiency level (> 22.7 μmol/L), and 9.2% of participants had concentrations considered extremely deficient (< 11 μmol/L) [28–30] (data not shown). For α-tocopherol, severe overt deficiency has been reported at 11.6 μmol/L [28] and levels > 27.5 μmol/L have been considered optimum to reduce the risk associated with ischaemic heart disease (IHD) [29]. More than two fifths of participants (40.5%) had plasma alpha tocopherol concentrations recommended for optimum health (> 27.5 μmol/L). Almost all participants (99.7%) had alpha tocopherol concentrations in excess of the overt deficiency value of 11.6 μmol/L (data not shown).

Optimum recommended concentrations to reduce IHD risk have previously been proposed for retinol (≥ 2.2 μmol/L) and β-carotene (> 0.4 μmol/L) [28–30]. Over half of the participants (54.5%) had plasma retinol concentrations ≥ 2.2 μmol/L and over one third (36.2%) had a β-carotene concentration > 0.4 μmol/L.

### Cardiovascular disease outcome

**Fig. 1:**
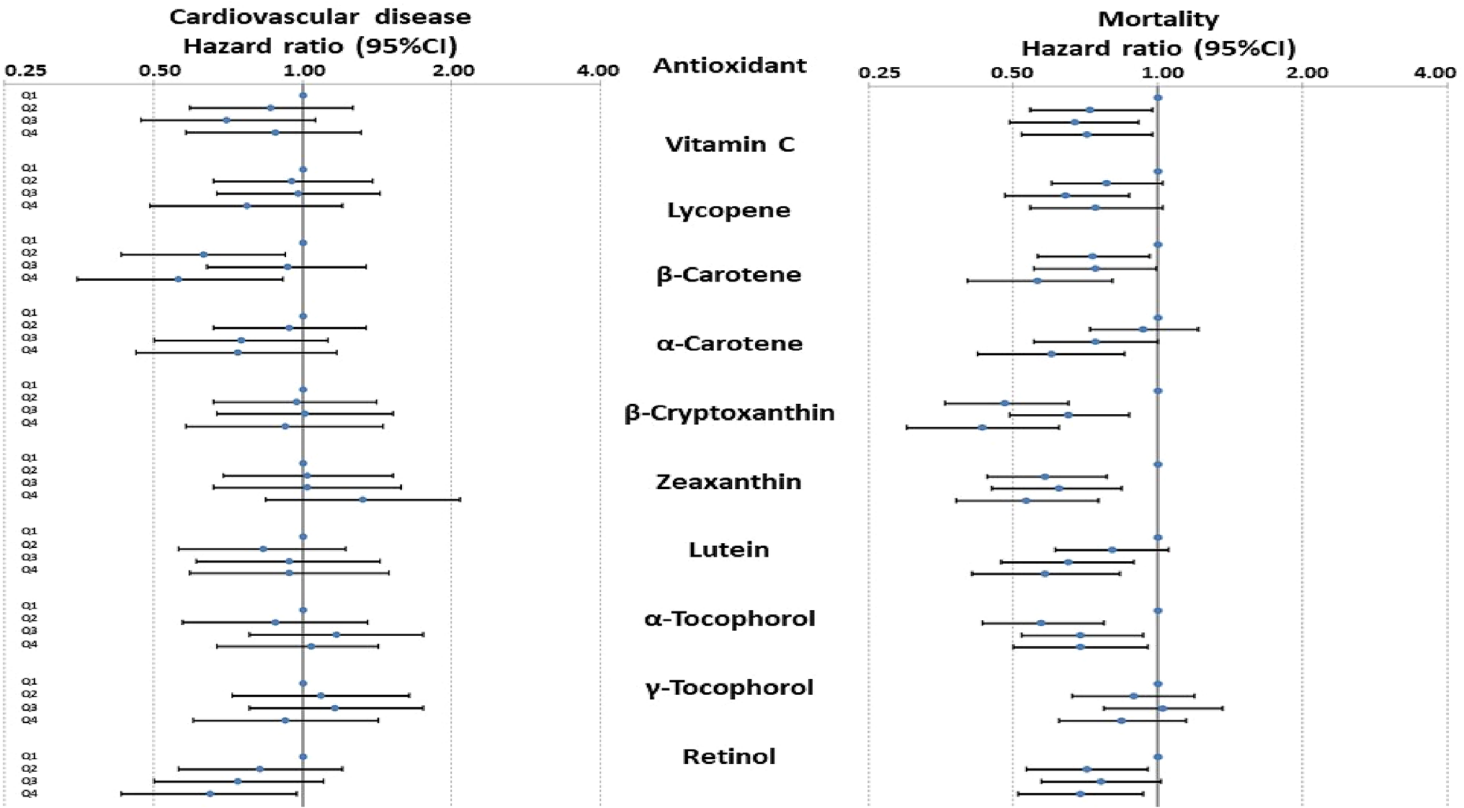
Hazard ratios for death from all causes and for a cardiovascular event by quarters of antioxidant levels, adjusting for age, country, diabetes, alcohol and smoking status, systolic blood pressure, body mass index, total cholesterol, high density lipoprotein cholesterol, triglycerides, height, social class and physical activity level

With the exception of both α- and γ-tocopherol, lower levels of all antioxidants were detected in men who experienced a CVD outcome, although this was not statistically significant for zeaxanthin and retinol (Table 2). Hazard ratios in a model minimally adjusted for age and country, showed a significant reduction in risk across quarters for alpha and beta carotene, vitamin C, lycopene, α-tocopherol and mean SAS (*p* < 0.05; Table 3). Following further adjustment for SBP, diabetes, BMI, cholesterol, HDL cholesterol, triglycerides, height, total physical activity, alcohol consumption, smoking and socioeconomic status, significant reductions across quarters of cardiovascular event risk were only observed for retinol, with α- and β-carotene falling just short of the significance threshold (*p* < 0.05; Table 4, Fig. 1). The mean SAS divided in quarters showed no significant association with CVD outcomes in the fully adjusted model (Table 4). Associations with CVD outcomes were little altered by the omission of total cholesterol, HDL cholesterol, triglycerides, and systolic blood pressure with the exception of the attenuated effect on retinol that no longer remained significant (Supplementary Tables 3a and 3b).

**Table 2:** Geometric mean antioxidant levels and interquartile range (IQR) for cardiovascular disease outcomes and all-cause mortality for 6961 men from Northern Ireland and France who were free of cardiovascular disease at entry

Antioxidant	CVD outcome				All-Cause Mortality		
μmol/L	All (*n* = 6961)	No (*n* = 6747)	Yes (*n* = 214)	*p* value	No (*n* = 6545)	Yes (*n* = 358)	*p* value
Vitamin C	33.8 (24.8–59.2)	33.9 (25.0–59.2)	29.8 (19.4–57.8)	0.045	34.4 (25.5–59.4)	24.1 (15.1–55.0)	< 0.001
Lycopene	0.67 (0.42–1.34)	0.67 (0.42–1.35)	0.52 (0.31–1.09)	0.001	0.68 (0.43–1.35)	0.48 (0.27–1.08)	< 0.001
β-Carotene	0.29 (0.17–0.51)	0.29 (0.17–0.51)	0.22 (0.11–0.39)	< 0.001	0.29 (0.18–0.52)	0.20 (0.11–0.40)	< 0.001
α-Carotene	0.11 (0.07–0.19)	0.11 (0.07–0.19)	0.08 (0.05–0.15)	< 0.001	0.11 (0.07–0.20)	0.08 (0.05–0.15)	< 0.001
β-Cryptoxanthin	0.70 (0.04–0.10)	0.07 (0.04–0.10)	0.06 (0.04–0.09)	0.008	0.07 (0.04–0.11)	0.05 (0.03–0.08)	< 0.001
Zeaxanthin	0.04 (0.03–0.06)	0.04 (0.03–0.06)	0.04 (0.03–0.06)	0.125	0.04 (0.03–0.06)	0.04 (0.02–0.05)	< 0.001
Lutein	0.21 (0.16–0.31)	0.22 (0.16–0.31)	0.19 (0.14–0.30)	0.006	0.22 (0.16–0.31)	0.17 (0.12–0.27)	< 0.001
α-Tocophorol	26.0 (22.2–30.4)	25.9 (22.1–30.4)	26.8 (22.5–31.5)	0.059	26.0 (22.3–30.5)	24.6 (20.5–29.8)	< 0.001
γ-Tocophorol	2.35 (1.88–2.95)	2.35 (1.88–2.95)	2.40 (1.93–2.96)	0.375	2.35 (1.88–2.95)	2.31 (1.84–2.86)	0.422
Retinol	2.24 (1.92–2.64)	2.24 (1.92–2.64)	2.22 (1.87–2.59)	0.525	2.24 (1.93–2.64)	2.15 (1.83–2.57)	0.006

**Table 3:** Minimally adjusted for age and country Cox Proportional Hazard Ratios (HR) by quartile for cardiovascular disease outcomes and all-cause mortality

	CVD outcomes				All-cause mortality			
	Q2 HR (95% CI)	Q3 HR (95% CI)	Q4 HR (95% CI)	*P*	Q2 HR (95% CI)	Q3 HR (95% CI)	Q4 HR (95% CI)	*p*
Vitamin C	0.77 (0.54–1.11)	0.59 (0.40–0.87)	0.72 (0.50–1.04)	0.04	0.57 (0.43–0.75)	0.49 (0.37–0.66)	0.51 (0.39–0.68)	< 0.001
Lycopene	0.86 (0.61–1.23)	0.79 (0.55–1.13)	0.56 (0.38–0.85)	0.006	0.69 (0.53–0.90)	0.53 (0.40–0.71)	0.51 (0.38–0.68)	< 0.001
β-Carotene	0.59 (0.41–0.85)	0.77 (0.55–1.09)	0.40 (0.26–0.62)	< 0.001	0.59 (0.46–0.77)	0.55 (0.42–0.72)	0.36 (0.26–0.49)	< 0.001
α-Carotene	0.85 (0.60–1.20)	0.64 (0.44–0.93)	0.54 (0.36–0.82)	0.001	0.79 (0.62–1.02)	0.56 (0.42–0.74)	0.40 (0.29–0.56)	< 0.001
β-Cryptoxanthin	0.85 (0.60–1.23)	0.81 (0.56–1.18)	0.67 (0.45–1.01)	0.06	0.39 (0.29–0.52)	0.47 (0.36–0.62)	0.30 (0.22–0.41)	< 0.001
Zeaxanthin	0.97 (0.66–1.41)	0.87 (0.57–1.31)	1.02 (0.68–1.54)	0.97	0.48 (0.36–0.63)	0.45 (0.34–0.60)	0.36 (0.26–0.49)	< 0.001
Lutein	0.77 (0.53–1.13)	0.77 (0.51–1.16)	0.78 (0.51–1.19)	0.27	0.67 (0.51–0.87)	0.48 (0.36–0.65)	0.40 (0.29–0.55)	< 0.001
α-Tocophorol	0.84 (0.56–1.28)	1.21 (0.83–1.77)	1.32 (0.91–1.91)	0.049	0.53 (0.40–0.70)	0.65 (0.49–0.85)	0.62 (0.47–0.81)	0.002
γ-Tocophorol	1.17 (0.79–1.73)	1.25 (0.85–1.84)	1.14 (0.77–1.69)	0.50	0.83 (0.63–1.10)	0.88 (0.67–1.16)	0.78 (0.59–1.04)	0.13
Retinol	0.81 (0.56–1.19)	0.87 (0.60–1.26)	0.85 (0.58–1.23)	0.45	0.71 (0.54–0.94)	0.75 (0.57–0.98)	0.77 (0.58–1.01)	0.07
Mean SAS	0.77 (0.56–1.08)	0.68 (0.48–0.97)	0.67 (0.47–0.96)	0.02	0.52 (0.41–0.67)	0.36 (0.27–0.48)	0.37 (0.28–0.49)	< 0.001

THR indicated for quarters 2, 3 and 4 are relative to the reference quarter 1

*Q* quarter, *HR* hazard ratio, *CI* confidence intervals, *Mean SAS* mean standardised antioxidant score, *p* *p* for trend

**Table 4:** Adjusted Cox Proportional Hazard Ratios (HR) by quartile for cardiovascular disease outcomes and all-cause mortality

	CVD outcomes				All-cause mortality			
	Q2 HR (95% CI)	Q3 HR (95% CI)	Q4 HR (95% CI)	*p*	Q2 HR (95% CI)	Q3 HR (95% CI)	Q4 HR (95% CI)	*p*
Vitamin C	0.85 (0.59–1.24)	0.69 (0.46–1.03)	0.86 (0.58–1.26)	0.27	0.69 (0.52–0.92)	0.63 (0.46–0.84)	0.66 (0.49–0.89)	0.003
Lycopene	0.93 (0.64–1.34)	0.95 (0.65–1.38)	0.73 (0.47–1.14)	0.24	0.77 (0.59–1.00)	0.63 (0.47–0.85)	0.70 (0.51–0.96)	0.005
β-Carotene	0.61 (0.42–0.90)	0.90 (0.62–1.30)	0.54 (0.34–0.86)	0.06	0.71 (0.54–0.93)	0.71 (0.53–0.95)	0.53 (0.37–0.74)	< 0.001
α-Carotene	0.92 (0.65–1.32)	0.73 (0.49–1.09)	0.71 (0.45–1.12)	0.07	0.92 (0.71–1.20)	0.72 (0.53–0.96)	0.56 (0.40–0.80)	0.001
β-Cryptoxanthin	0.94 (0.64–1.36)	0.99 (0.67–1.47)	0.86 (0.56–1.32)	0.57	0.46 (0.35–0.62)	0.63 (0.47–0.83)	0.41 (0.29–0.57)	< 0.001
Zeaxanthin	1.02 (0.69–1.51)	1.00 (0.65–1.54)	1.26 (0.81–1.95)	0.36	0.58 (0.43–0.77)	0.60 (0.44–0.81)	0.50 (0.36–0.71)	< 0.001
Lutein	0.81 (0.55–1.19)	0.91 (0.59–1.38)	0.90 (0.57–1.41)	0.73	0.79 (0.60–1.04)	0.63 (0.46–0.86)	0.56 (0.40–0.79)	< 0.001
α-Tocophorol	0.85 (0.56–1.30)	1.13 (0.75–1.68)	0.99 (0.64–1.53)	0.72	0.56 (0.42–0.75)	0.68 (0.51–0.91)	0.66 (0.48–0.91)	0.02
γ-Tocophorol	1.06 (0.70–1.59)	1.14 (0.76–1.70)	0.90 (0.59–1.38)	0.69	0.87 (0.65–1.17)	1.01 (0.76–1.35)	0.83 (0.61–1.13)	0.43
Retinol	0.82 (0.56–1.21)	0.76 (0.52–1.12)	0.65 (0.43–0.98)	0.04	0.70 (0.53–0.94)	0.74 (0.56–0.99)	0.68 (0.50–0.91)	0.02
Mean SAS	0.83 (0.59–1.17)	0.79 (0.55–1.14)	0.76 (0.51–1.13)	0.16	0.61 (0.47–0.79)	0.46 (0.34–0.62)	0.50 (0.37–0.68)	< 0.001

The HR indicated for quarters 2, 3 and 4 are relative to the reference quarter 1. The Cox Proportional HRs are adjusted for age, country, diabetes, alcohol and smoking status, systolic blood pressure, body mass index, total cholesterol, high density lipoprotein cholesterol, triglycerides, height, social class and physical activity level

*Q* quarter, *HR* hazard ratio, *CI* confidence intervals, *Mean SAS* mean standardised antioxidant score, *p* *p* for trend

### All-cause mortality outcome

Men who died during follow-up were on average older (55.8 years versus 54.8 years), more likely to be from NI (31.8% versus 25.2%) and twice as likely to have diabetes (6.5% versus 3.1%) than men who survived. They also had higher mean SBP (139 mmHg versus 133 mmHg), higher median triglyceride levels (1.31 g/L versus 1.22 g/L) and were less physically active (median METs 73.6 versus 83.3). Furthermore, they were significantly more likely to be from the low social class (37.3% versus 23.4%), to consume fewer daily FVJ portions (median 2.14 versus 2.43) and have higher levels of both smoking and alcohol consumption (*p* < 0.001).

There were lower levels of all antioxidants associated with all-cause mortality, and only that of γ-tocopherol was not significant (Table 2). Similarly fully adjusted hazard ratios showed statistically significant reductions for all antioxidants in association with all-cause mortality, with the exception of γ-tocopherol (Fig. 1; Tables 3 and 4). Daily FVJ portions were initially significant when fitted with other confounders within the adjusted model but was no longer significant when the mean *Z* score was included. The mean SAS showed significantly reduced risk across quarters for all-cause mortality in minimally and fully adjusted models (Tables 3 and 4; *p* < 0.001). Lack of linearity was observed for the associations between mortality and α-tocopherol, β-cryptoxanthin, zeaxanthin and SAS. Similar adjusted Cox proportional HR by quartile for smoking-associated cancer and all neoplasm-related mortality according to ICD Ninth Revision codes are presented in Supplementary Table 4.

No significant deviations from the tests of the hazard proportionality assumption in the Cox model using Schoenfeld residuals were detected, for either CVD events or all-cause mortality. There was no evidence of significant interaction between country and antioxidants in any of the models for CVD events or all-cause mortality following correction for multiple testing.

## Discussion

In this study, we observed associations between lower concentrations of serum micronutrients vitamin C, retinol, α-tocopherol, and six carotenoids, including lycopene, α-carotene, β-carotene, β-cryptoxanthin, zeaxanthin and lutein, and all-cause mortality over a 10-year follow-up period, using data from a large cohort of 9709 predominantly white, middle-aged men who were free from CVD at the time of recruitment in NI and France. Furthermore, higher plasma retinol concentrations were also significantly associated with a lower risk of CVD events after adjustment for potential confounding variables.

Oxidative stress and systemic inflammatory damage have been implicated in the aetiology of several chronic diseases, including CVD [2–4], with nutritional influences previously estimated to contribute to almost 45% of all cardiometabolic deaths in the United States in 2012 [5–10]. Previously, we reported low plasma retinol independent of carotenoid levels in association with CVD risk, in a nested case control study of PRIME participants after 5-year follow-up [5]. Retinol is partly replaced by its major precursor, β-carotene, although conversion is poor and inconsistent [31]. The relatively moderate association of plasma retinol in the present evaluation may be limited by the complexities of plasma retinol determination, dietary influences and vitamin A status, especially given the influences of hepatic homeostasis and reported age-dependent variation in middle-aged westernised males, mean age at sampling/recruitment of 55 years, and subsequent CVD and all-cause mortality outcomes over the following decade [5, 31]. All other evaluated micronutrients were not significantly associated with reduced CVD risk, following adjustment for potential confounders. These findings largely support previous observational reports of inverse associations between all-cause mortality, cardiovascular risk and serum micronutrient status [5, 32, 33], supported by higher fruit and vegetable consumption and antioxidant intake [34–36].

Carotenoids are lipophilic phytochemicals represented by two sub-classes based upon their polarity: non-polar carotenes include α-carotene, β-carotene and lycopene while polar xanthophylls include lutein, zeaxanthin and β-cryptoxanthin. The individual carotenoid properties are dependent on their unique functional group and the conjugated double-bonding present [37]. Carotenoids have been reported to reduce the detrimental impact of oxidative stress-induced diseases including CVD, given the antioxidant and anti-inflammatory properties that result from their chemical structure and interaction with biological membranes [38]. Interestingly, the reduced risk of CVD events and all-cause mortality associated with antioxidants identified from observational epidemiological studies has not been observed in supplementation trials, some of which have not only failed to show any health benefits but have reported unanticipated harmful effects [14]. Huang and colleagues also recently identified inverse associations between serum β-carotene and all-cause mortality, including CVD related deaths in a prospective serological analysis of 29,103 men in the ATBC study (Alpha-Tocopherol, Beta-Carotene Cancer Prevention) over 31 years of follow-up that assessed 23,796 deaths [14]. Randomised supplementation trials have been largely limited to single antioxidant studies, in contrast to observational epidemiological investigations of plasma antioxidant levels or dietary intake of foods rich in these nutrients. The dietary origins of these antioxidants (fruits, vegetables, berries, etc.) also contain additional natural compounds that may enhance the micronutrient properties that are difficult to replicate in the context of supplementation studies [10]. Previous investigations have highlighted that individuals with low micronutrient status are more likely to benefit from randomised supplementation trials [15]. Reduced all-cause mortality was reported previously following supplementation in nutritionally deficient individuals with low plasma antioxidant levels, but excess levels in sufficient individuals may not provide additional benefit and result in potentially toxic effects [15–17].

β-Carotene is one of the most widely studied carotenoids given its abundance in fruit and vegetables and its pro-vitamin A activity. The beneficial antioxidant properties of β-carotene include improved immunological function through enhanced lymphocyte proliferation that reduces LDL susceptibility to oxidative modification [39]. Nevertheless, the benefits associated with dietary consumption appear to be lost when it is provided by pharmacological supplementation, which in turn may result in harmful effects in some sub-populations, such as smokers, where increased incidence of lung cancer and CVD have been reported previously [40]. A possible explanation for the lack of association between serum micronutrient status and CVD beyond retinol, may result from the lower number of recorded CVD events than deaths in PRIME participants (440 versus 538), and as blood samples were not available for all PRIME participants, the number of CVD events included in the analysis was further limited, which may have reduced statistical power. Survival bias may also have been a factor, as men with lower plasma micronutrient concentrations would be more likely to have died prematurely during the 10-year follow-up period and therefore were less likely to have recorded a CVD event within the specified time period, given the resultant death.

Higher plasma concentrations of vitamin C, retinol, α-tocopherol and all carotenoids were associated with reduced all-cause mortality among the middle-aged male participants of the PRIME study. In keeping with the findings reported here, a recent systematic review and meta-analyses identified no associations between α-carotene, lycopene, β-cryptoxanthin, lutein and zeaxanthin with cardiovascular outcomes [10]. Aune and colleagues meta-analyses reported associations between α-carotene, lycopene, β-cryptoxanthin and mortality similar to our findings, although in contrast, they failed to detect associations between lutein and zeaxanthin and overall mortality [10]. The limited statistical power associated with the analysis of incident CVD cases reported in the current study and the small number of studies available for meta-analyses previously, should be considered in the context of the findings reported.

In contrast to the other micronutrients evaluated in the current study, plasma γ-tocopherol levels were not associated with all-cause mortality. Evaluation of the effects of vitamin E has predominately focused on α-tocopherol, with evidence specific to γ-tocopherol and health-related outcomes, less commonly studied [41]. Previous studies, such as the British National Diet and Nutrition Survey of those aged 65 years and over, also found no evidence of association between plasma γ-tocopherol and overall mortality (HR 0.96, *p* = 0.3) [42]. In contrast, the Multiethnic Cohort Study evaluated 1489 deaths reported in 8365 participants and serum γ-tocopherol was positively associated with all-cause, cancer and CVD mortality in adjusted models [32].

The potential benefits of vitamin C on mortality have been previously reported [43] and several potential mechanisms proposed, including a protective effect on the vascular endothelium, preventing uncontrolled proliferation of vascular smooth muscle cells and inhibition of pro-inflammatory cytokines and adhesion molecules central to the atherogenic processes [44]. Previous studies have also reported associations between higher carotenoid concentrations and lower cancer [7, 45], dementia [46] and CVD risk [5–10] and between carotenoids and markers of inflammation and oxidative stress [13].

These findings have important implications for future antioxidant research, given the variation in effects on all-cause mortality observed, highlighting the importance of measuring individual micronutrient levels. Antioxidants possess different molecular properties and biological activities that confer variable health benefits and the non-linear relationship observed for some antioxidants suggest a beneficial range that may be more readily achieved through dietary interventions as opposed to high-dose supplementation [15]. Micronutrients are unlikely to act independently in their reduction of mortality risk but reflect specific food groups, such as fruit and vegetables, that are associated with improved health outcomes [47]. Further assessment of the interactions, optimal concentrations and potential health benefits of antioxidants will improve the design of dietary interventions and identify those most likely to benefit.

### Strengths and limitations of this study

The major strengths of our study were the large number of well-characterised participants with prospective follow-up over 10 years and clinically validated cardiovascular events. Many observational studies are restricted by small sample size and/or short follow-up duration, limiting the reliability of the associations reported. Associations were based only on those men free from cardiovascular events at study entry. In addition, the micronutrient plasma measurements were quantified using standardised and validated laboratory methods, and so provided an objective determinant, independent of dietary intake estimates recalled over a period of time. Assessment of antioxidant concentrations as continuous variables, identified similar associations to the evaluation of antioxidants by quartiles but the latter is more amenable to the identification of non-linear relationships, where they exist. Alpha-carotene and lutein were the only antioxidants that demonstrated a dose–response relationship with all-cause mortality, with higher concentrations associated with lower mortality. Retinol was the only antioxidant significantly associated with CVD events demonstrating a linear relationship with reduced risk with higher concentrations. A mean SAS offers an opportunity to assess the cumulative effects of antioxidant concentrations on CVD outcomes and all-cause mortality. Although a combined total antioxidant score was more strongly associated with all-cause mortality than individual antioxidants, the effects observed were not substantially different than for individual antioxidants alone.

Our study has several limitations. We tested multiple hypotheses and, as such, our results must be interpreted with caution as we made no correction for multiple testing in the analyses presented in Tables 2, 3 and 4, although several of the reported associations would remain significant even if tested at the *p* < 0.001 level. Despite controlling for the most important covariates, additional residual confounding may exist. Furthermore, additional dietary factors not considered within the analyses may be associated with antioxidant levels. Moreover, the sensitivity of nutritional biomarkers is somewhat limited by the influences of individual genetic variation associated with dietary intake and nutrient metabolism, a complex multistage process that includes ingestion, digestion, absorption and transportation in the blood [48, 49]. The categorical analyses by quartiles depends on certain parametric modelling assumptions (proportional hazards, linearity, etc.), although analyses of antioxidant levels as continuous variables demonstrated similar levels of significance (data not shown). In addition, while we evaluated daily FVJ consumption, the use of FFQ data may lead to an overestimation of the intakes of some healthy foods, which may impact the interpretation of the associations observed, although this had little effect on the associated hazard ratio estimates. Furthermore, we used a single measure of plasma antioxidant levels which may not necessarily reflect long-term nutritional status against CVD and all-cause mortality outcomes. Previous analysis of The Third National Health and Nutrition Examination Survey (NHANES III) investigated 4225 deaths in 16,008 participants over a median 14.2 years follow-up period and reported that a single serum measurement likely represents a reliable estimate of long-term antioxidant status [15]. Additionally, associations between micronutrient status and fruit and vegetable intake were strong, supporting the plasma concentration as an appropriate representative measure. Although we examined the independent and cumulative effects of ten antioxidants on cardiovascular events and all-cause mortality, potential associations with other nutrients such as omega-3-fatty acids, vitamin D, the B-vitamins and selenium were beyond the scope of this study. Finally, our study results apply to predominantly white European middle-aged men who were initially free from CVD at the point of recruitment.

In summary, we have shown that higher plasma concentrations of vitamin C, retinol, α-tocopherol and six carotenoids (α-carotene, β-carotene, β-cryptoxanthin, lutein, lycopene, zeaxanthin) were associated with a lower risk of all-cause mortality in middle-aged men. Furthermore, higher levels of retinol were associated with reduced risk of CVD events. Our findings are of major public health significance as the search for improved understanding of the modifiable risk factors associated with cardiovascular events and all-cause mortality continues. Identifying individuals at future increased risk of developing CVD and detecting adverse risk at the earliest stages, may be most effective in delaying disease progression and preventing premature death. Our findings suggest that, at a population level, small, achievable shifts in micronutrient status could have a considerable impact on mortality and CVD risk. However, there is still insufficient evidence to establish a causal relationship between low antioxidant levels and all-cause mortality. Given the potential implications of nutritional status in the aetiology of multiple chronic conditions, dietary intervention warrants further investigation and may prove beneficial in reducing premature mortality, especially given the largely null findings from randomised control trails of antioxidant supplementation. Insufficient consideration for individual participant baseline nutrition levels within study inclusion criteria, may represent a major concern for randomised trial design. Our findings suggest individuals deficient in specific micronutrients may derive greater benefit from dietary or supplement interventions than sufficient individuals.

## Supplementary Information

Below is the link to the electronic supplementary material.

Supplementary file1 (DOCX 44 KB)
