# Supplementary material for: Association of low plasma antioxidant levels with all-cause mortality and coronary events in healthy middle-aged men from France and Northern Ireland in the PRIME study
Source: Eur J Nutr. 2020 Dec 23;60(5):2631–41. doi: 10.1007/s00394-020-02455-2 (PMC8275518; doi:10.1007/s00394-020-02455-2)
Supplement: Supplementary file 1 — Supplementary file1 (DOCX 44 KB) [file 394_2020_2455_MOESM1_ESM.docx]

**Supplementary Table 1**. Quartile boundaries and geometric means for each antioxidant.

| **Antioxidant**  **(μmol/L)** | **Quarter 1**  **Range GM** | | **Quarter 2**  **Range GM** | | **Quarter 3**  **Range GM** | | **Quarter 4**  **Range GM** | |
| --- | --- | --- | --- | --- | --- | --- | --- | --- |
| Vitamin C | <24.00 | 10.17 | 24.0-41.99 | 32.88 | 42.00-58.87 | 49.75 | >58.87 | 73.62 |
| Lycopene | <0.413 | 0.147 | 0.413-0.811 | 0.603 | 0.812-1.325 | 1.036 | >1.325 | 2.05 |
| β-Carotene | <0.168 | 0.091 | 0.168-0.299 | 0.227 | 0.300-0.503 | 0.386 | >0.503 | 0.792 |
| α-Carotene | <0.064 | 0.032 | 0.064-0.116 | 0.089 | 0.117-0.191 | 0.149 | >0.191 | 0.293 |
| β-Cryptoxanthin | <0.041 | 0.026 | 0.041-0.064 | 0.052 | 0.065-0.102 | 0.081 | >0.102 | 0.154 |
| Zeaxanthin | <0.032 | 0.023 | 0.032-0.045 | 0.039 | 0.046-0.061 | 0.052 | >0.061 | 0.077 |
| Lutein | <0.153 | 0.108 | 0.153-0.220 | 0.185 | 0.220-0.305 | 0.258 | >0.305 | 0.395 |
| α-Tocophorol | <22.15 | 18.84 | 22.15-26.05 | 24.16 | 26.06-30.49 | 28.08 | >30.49 | 35.62 |
| γ-Tocophorol | <1.871 | 1.53 | 1.871-2.351 | 2.11 | 2.352-2.946 | 2.62 | >2.946 | 3.59 |
| Retinol | <1.913 | 1.61 | 1.913-2.251 | 2.09 | 2.252-2.636 | 2.43 | >2.636 | 3.06 |

GM = geometric mean

**Supplementary Table 2.** Correlation coefficients between daily fruit, vegetable and juice consumption and antioxidant status.

|  | Vitamin C | Lycopene | β-Carotene | α-Carotene | β-Cryptoxanthin | Zeaxanthin | Lutein | α-Tocophorol | γ-Tocophorol | Retinol |
| --- | --- | --- | --- | --- | --- | --- | --- | --- | --- | --- |
| Vitamin C | 1.00 | .18^‡^ | .27^‡^ | .23^‡^ | .40^‡^ | .30^‡^ | .25^‡^ | .09^‡^ | .03^*^ | .01 |
| Lycopene |  | 1.00 | .70^‡^ | .67^‡^ | .39^‡^ | .32^‡^ | .30^‡^ | .11^‡^ | .10^‡^ | -.01 |
| β-Carotene |  |  | 1.00 | .86^‡^ | .51^‡^ | .44^‡^ | .45^‡^ | .13^‡^ | .10^‡^ | -.04^‡^ |
| α-Carotene |  |  |  | 1.00 | .48^‡^ | .44^‡^ | .49^‡^ | .13^‡^ | .09^‡^ | .01 |
| β-Cryptoxanthin |  |  |  |  | 1.00 | .68^‡^ | .53^‡^ | .29^‡^ | .18^‡^ | .04 ^‡^ |
| Zeaxanthin |  |  |  |  |  | 1.00 | .87^‡^ | .39^‡^ | .22^‡^ | .13^‡^ |
| Lutein |  |  |  |  |  |  | 1.00 | .36^‡^ | .21^‡^ | .13^‡^ |
| α-Tocophorol |  |  |  |  |  |  |  | 1.00 | .48^‡^ | .40^‡^ |
| γ-Tocophorol |  |  |  |  |  |  |  |  | 1.00 | .25^‡^ |
| Retinol |  |  |  |  |  |  |  |  |  | 1.00 |
|  |  |  |  |  |  |  |  |  |  |  |
| Daily fruit consumption | .26^‡^ | .12^‡^ | .17^‡^ | .17^‡^ | .35^‡^ | .19^‡^ | .16^‡^ | .03^*^ | -.01 | -.03^‡^ |
| Daily vegetable consumption | .16^‡^ | .13^‡^ | .17^‡^ | .17^‡^ | .17^‡^ | .20^‡^ | .20^‡^ | .06^‡^ | .02 | .01 |
| Daily fruit and vegetable consumption | .27^‡^ | .16^‡^ | .22^‡^ | .22^‡^ | .34^‡^ | .24^‡^ | .23^‡^ | .06^‡^ | .01 | -.02 |
| Daily juice consumption | .16^‡^ | .02 | .02^*^ | .00 | .12^‡^ | .03^*^ | -.03^‡^ | .04^‡^ | .05^‡^ | .03^‡^ |
| Daily fruit, vegetable and juice consumption | .30^‡^ | .15^‡^ | .21^‡^ | .20^‡^ | .35^‡^ | .24^‡^ | .20^‡^ | .06^‡^ | .02 | -.01 |

All biomarkers were log-transformed prior to analysis. ^*^P< 0.05, ^‡^P< 0.01 (2-tailed).

**Supplementary Table 3a**. Adjusted Cox Proportional Hazard Ratios (HR) by quartile for cardiovascular disease outcomes and all-cause mortality. Note the HR indicated for quarters 2, 3 and 4 are relative to the reference quarter 1. The Cox Proportional HRs are adjusted for age, country, diabetes, alcohol and smoking status, systolic blood pressure, body mass index, height, social class and physical activity level.

|  | **CVD Outcomes** | | | | **All-cause mortality** | | | |
| --- | --- | --- | --- | --- | --- | --- | --- | --- |
|  | **Q2 HR (95% CI)** | **Q3 HR (95% CI)** | **Q4 HR (95% CI)** | **P** | **Q2 HR (95% CI)** | **Q3 HR (95% CI)** | **Q4 HR (95% CI)** | **P** |
| Vitamin C | 0.86 (0.59-1.25) | 0.69 (0.46-1.03) | 0.86 (0.58-1.26) | 0.26 | 0.67 (0.50-0.89) | 0.62 (0.46-0.83) | 0.66 (0.49-0.88) | 0.002 |
| Lycopene | 0.94 (0.65-1.36) | 0.97 (0.67-1.41) | 0.78 (0.50-1.21) | 0.36 | 0.76 (0.58-0.99) | 0.64 (0.48-0.85) | 0.70 (0.51-0.96) | 0.005 |
| β-Carotene | 0.65 (0.45-0.95) | 0.94 (0.66-1.36) | 0.59 (0.37-0.93) | 0.10 | 0.69 (0.53-0.90) | 0.72 (0.54-0.95) | 0.53 (0.38-0.74) | <0.001 |
| α-Carotene | 0.96 (0.67-1.36) | 0.75 (0.51-1.12) | 0.76 (0.48-1.19) | 0.13 | 0.92 (0.71-1.18) | 0.71 (0.53-0.95) | 0.56 (0.40-0.80) | <0.001 |
| β-Cryptoxanthin | 0.99 (0.68-1.45) | 1.08 (0.73-1.60) | 0.91 (0.60-1.40) | 0.81 | 0.47 (0.36-0.63) | 0.62 (0.47-0.82) | 0.40 (0.28-0.56) | <0.001 |
| Zeaxanthin | 1.08 (0.73-1.60) | 1.08 (0.70-1.65) | 1.38 (0.90-2.13) | 0.17 | 0.57 (0.43-0.76) | 0.58 (0.43-0.79) | 0.49 (0.35-0.69) | <0.001 |
| Lutein | 0.86 (0.58-1.27) | 0.97 (0.64-1.48) | 1.00 (0.64-1.56) | 0.90 | 0.79 (0.60-1.04) | 0.62 (0.46-0.85) | 0.55 (0.40-0.78) | <0.001 |
| α-Tocophorol | 0.89 (0.59-1.36) | 1.26 (0.86-1.87) | 1.27 (0.86-1.87) | 0.09 | 0.57 (0.43-0.76) | 0.69 (0.52-0.91) | 0.65 (0.49-0.86) | 0.005 |
| γ-Tocophorol | 1.13 (0.75-1.70) | 1.25 (0.84-1.86) | 1.06 (0.71-1.59) | 0.69 | 0.86 (0.65-1.15) | 0.96 (0.72-1.27) | 0.79 (0.59-1.06) | 0.20 |
| Retinol | 0.85 (0.58-1.24) | 0.81 (0.55-1.19) | 0.73 (0.49-1.08) | 0.12 | 0.69 (0.52-0.92) | 0.72 (0.54-0.96) | 0.67 (0.50-0.89) | 0.01 |
| Mean SAS | 0.88 (0.63-1.24) | 0.86 (0.60-1.23) | 0.86 (0.59-1.27) | 0.42 | 0.61 (0.47-0.78) | 0.46 (0.34-0.61) | 0.50 (0.37-0.67) | <0.001 |

Q: quarter; HR: Hazard Ratio; CI: confidence intervals; Mean SAS: mean standardised antioxidant score; P: P for trend.

**Supplementary Table 3b**. Adjusted Cox Proportional Hazard Ratios (HR) by quartile for cardiovascular disease outcomes and all-cause mortality. Note the HR indicated for quarters 2, 3 and 4 are relative to the reference quarter 1. The Cox Proportional HRs are adjusted for age, country, diabetes, alcohol and smoking status, body mass index, height, social class and physical activity level.

|  | **CVD Outcomes** | | | | **All-cause mortality** | | | |
| --- | --- | --- | --- | --- | --- | --- | --- | --- |
|  | **Q2 HR (95% CI)** | **Q3 HR (95% CI)** | **Q4 HR (95% CI)** | **P** | **Q2 HR (95% CI)** | **Q3 HR (95% CI)** | **Q4 HR (95% CI)** | **P** |
| Vitamin C | 0.84 (0.58-1.21) | 0.66 (0.45-0.99) | 0.80 (0.55-1.18) | 0.15 | 0.67 (0.51-0.90) | 0.61 (0.46-0.82) | 0.65 (0.49-0.87) | 0.002 |
| Lycopene | 0.95 (0.66-1.37) | 0.96 (0.66-1.40) | 0.75 (0.48-1.16) | 0.26 | 0.76 (0.58-0.98) | 0.63 (0.47-0.84) | 0.71 (0.52-0.98) | 0.005 |
| β-Carotene | 0.64 (0.44-0.92) | 0.90 (0.63-1.30) | 0.55 (0.35-0.86) | 0.05 | 0.69 (0.53-0.90) | 0.71 (0.54-0.94) | 0.52 (0.37-0.73) | <0.001 |
| α-Carotene | 0.99 (0.69-1.40) | 0.77 (0.52-1.14) | 0.73 (0.47-1.14) | 0.10 | 0.91 (0.71-1.18) | 0.72 (0.54-0.96) | 0.56 (0.40-0.79) | <0.001 |
| β-Cryptoxanthin | 0.97 (0.66-1.40) | 0.99 (0.67-1.47) | 0.86 (0.56-1.31) | 0.55 | 0.47 (0.35-0.63) | 0.61 (0.46-0.81) | 0.39 (0.28-0.55) | <0.001 |
| Zeaxanthin | 1.07 (0.73-1.58) | 1.09 (0.71-1.67) | 1.34 (0.87-2.06) | 0.20 | 0.57 (0.43-0.76) | 0.58 (0.43-0.78) | 0.49 (0.35-0.68) | <0.001 |
| Lutein | 0.83 (0.57-1.23) | 0.96 (0.63-1.46) | 1.00 (0.64-1.56) | 0.88 | 0.78 (0.60-1.02) | 0.61 (0.45-0.83) | 0.55 (0.39-0.77) | <0.001 |
| α-Tocophorol | 0.93 (0.61-1.42) | 1.30 (0.88-1.92) | 1.35 (0.92-1.98) | 0.05 | 0.58 (0.43-0.77) | 0.70 (0.54-0.93) | 0.67 (0.51-0.88) | 0.01 |
| γ-Tocophorol | 1.16 (0.78-1.74) | 1.26 (0.85-1.88) | 1.10 (0.74-1.65) | 0.59 | 0.86 (0.65-1.15) | 0.95 (0.71-1.25) | 0.79 (0.59-1.05) | 0.18 |
| Retinol | 0.88 (0.60-1.29) | 0.87 (0.60-1.28) | 0.81 (0.54-1.20) | 0.30 | 0.71 (0.53-0.94) | 0.74 (0.56-0.98) | 0.71 (0.53-0.94) | 0.02 |
| Mean SAS | 0.88 (0.63-1.24) | 0.83 (0.58-1.19) | 0.84 (0.57-1.23) | 0.32 | 0.61 (0.48-0.79) | 0.45 (0.34-0.61) | 0.50 (0.37-0.67) | <0.001 |

Q: quarter; HR: Hazard Ratio; CI: confidence intervals; Mean SAS: mean standardised antioxidant score; P: P for trend.

**Supplementary Table 4**. Adjusted Cox Proportional Hazard Ratios (HR) by quartile for smoking-associated cancer and all neoplasm mortality. Note the HR indicated for quarters 2, 3 and 4 are relative to the reference quarter 1. The Cox Proportional HRs are adjusted for age, country, diabetes, alcohol and smoking status, systolic blood pressure, body mass index, total cholesterol, high density lipoprotein cholesterol, triglycerides, height, social class and physical activity level.

|  | **Smoking-associated cancer mortality** | | | | **All-neoplasm mortality** | | | |
| --- | --- | --- | --- | --- | --- | --- | --- | --- |
|  | **Q2 HR (95% CI)** | **Q3 HR (95% CI)** | **Q4 HR (95% CI)** | **P** | **Q2 HR (95% CI)** | **Q3 HR (95% CI)** | **Q4 HR (95% CI)** | **P** |
| Vitamin C | 0.48 (0.28-0.83) | 0.51 (0.30-0.88) | 0.24 (0.12-0.49) | <0.001 | 0.63 (0.43-0.91) | 0.56 (0.38-0.82) | 0.58 (0.40-0.86) | 0.003 |
| Lycopene | 0.74 (0.45-1.22) | 0.50 (0.28-0.90) | 0.63 (0.34-1.18) | 0.04 | 0.80 (0.57-1.12) | 0.54 (0.37-0.81) | 0.67 (0.44-1.00) | 0.008 |
| β-Carotene | 0.64 (0.39-1.06) | 0.47 (0.24-0.91) | 0.47 (0.24-0.91) | 0.007 | 0.71 (0.50-1.01) | 0.66 (0.45-0.97) | 0.52 (0.34-0.82) | 0.003 |
| α-Carotene | 0.61 (0.36-1.03) | 0.57 (0.32-1.00) | 0.48 (0.25-0.93) | 0.02 | 0.79 (0.56-1.11) | 0.69 (0.47-1.00) | 0.53 (0.34-0.83) | 0.003 |
| β-Cryptoxanthin | 0.32 (0.17-0.59) | 0.56 (0.32-0.95) | 0.33 (0.17-0.65) | 0.002 | 0.37 (0.25-0.55) | 0.55 (0.38-0.80) | 0.40 (0.26-0.61) | <0.001 |
| Zeaxanthin | 0.69 (0.41-1.16) | 0.58 (0.33-1.04) | 0.30 (0.14-0.63) | 0.001 | 0.55 (0.37-0.80) | 0.65 (0.44-0.96) | 0.45 (0.29-0.70) | 0.001 |
| Lutein | 0.71 (0.41-1.21) | 0.64 (0.36-1.15) | 0.43 (0.21-0.86) | 0.02 | 0.84 (0.59-1.20) | 0.60 (0.40-0.91) | 0.55 (0.35-0.87) | 0.004 |
| α-Tocophorol | 0.52 (0.29-0.93) | 0.72 (0.42-1.23) | 0.54 (0.29-1.00) | 0.10 | 0.61 (0.42-0.89) | 0.70 (0.49-1.02) | 0.62 (0.40-0.94) | 0.04 |
| γ-Tocophorol | 0.78 (0.45-1.34) | 0.81 (0.47-1.40) | 0.55 (0.30-1.01) | 0.08 | 0.90 (0.62-1.31) | 1.18 (0.83-1.69) | 0.65 (0.42-0.99) | 0.20 |
| Retinol | 0.72 (0.40-1.30) | 1.00 (0.58-1.73) | 0.73 (0.40-1.32) | 0.51 | 0.75 (0.51-1.10) | 0.93 (0.65-1.34) | 0.73 (0.49-1.09) | 0.26 |
| Mean SAS | 0.38 (0.22-0.64) | 0.45 (0.27-0.77) | 0.24 (0.12-0.48) | <0.001 | 0.54 (0.38-0.76) | 0.48 (0.33-0.70) | 0.43 (0.29-0.65) | <0.001 |

Q: quarter; HR: Hazard Ratio; CI: confidence intervals; Mean SAS: mean standardised antioxidant score; P: P for trend.
